# Treatment of Exposed Pulps

**Published:** 1877-07

**Authors:** F. C. Eddleman


### TREATMENT OF EXPOSED PULPS.

BY F. C. EDDLEMAN. D. D. S.

*Read before the Indiana State Dental Association.*

MR. PRESIDENT AND GENTLEMEN OF THE INDIANA STATE DENTAL ASSOCIATION :

In compliance with your action in convention last year and by request of your secretary I have the honor to present the following report on "Exposed Dental Pulps and Treatment."

I have chosen as a basis for this report thirty five cases beginning with the year 1876 and running through a period of several months of my daily practice. These are therefore not in any sense, selected with reference to either success or failure. I have chosen to make this selection from a previous years practice as being a more satisfactory test of success than those more recently treated.

Of the whole number treated, I am lead to believe that only three failures have resulted. It is true that my information is not of a positive character for I have to depend to some extent on my patients for a report.

Of two of the failures I can give no satisfactory account, even to my own mind.

The third failure, which we will denominate thirty-five of the whole series, is capable of a very easy solution. Its history is as follows, viz : Mrs. F—— of about thirty-five years of age, in figure rather slight, of phthisical diathesis ; occupation, mantua maker; called February 12th for an examination, and directed special attention to the left inferior first bicuspid; found the dental pulp fully exposed and somewhat inflamed—patient complaining of pain in that organ. At this sitting gave some preliminary treatment, and dismissed the patient after making an appointment for a subsequent call. At the second sitting two days later found patient more comfortable, and after the usual preparation of cavity of decay, removal of dressing, etc., having applied the rubber dam, protected the pulp in the usual manner with liquid gutta percha, and then applied oxy-chloride of zinc to close up the breach into the pulp cell, filled the cavity of decay with amalgam. Twenty days later, patient called saying that she had suffered more or less almost continuously, ever since her course of treatment; the character of the pain being the same as before.

At this sitting removed the plug, dressing and all but found everything in good condition except that the tooth was somewhat sensitive to pressure and to slight rappings upon it. The pulp was no more sensitive than a healthy organ, which in fact I think it was. I then advised devitalization of the pulp for fear that my suspicions of true condition might not be correct. I then made application arsenious acid or nerve paste and dismissed my patient after fixing a time for a subsequent call. Her time being well employed in her business she this time remained away for ten days at which time she again called with the same report a complaint of "the same old pain" using her language. Again removed dressing and found the pulp possessing some vitality at the extremity of root but removed it and filled root with gold,—and crown cavity with Hills stopping and dismissed patient with request that she report any unrest that she might notice, which she did in a few days, say five or six. At this setting decided to extract, but she objected to the loss of the tooth. I then decided to extract and replant to which she readily consented, I then proceeded to extract which was done with difficulty on account of a large exostosis about the apex of the root. This was dressed off, the tooth still remaining in the forceps, and the organ returned to its socket, and the patient dismissed. To my mind the exostosis upon the tooth sufficiently explains the whole trouble in this case.

As to treatment in the case of simple exposure, I remark that I am disposed to adopt as my theory protection and that only. The substance used for this purpose must possess certain qualities which are indispensable to success, viz: 1 inertness; 2 cohesiveness of its own particles; 3 non-conductivity and adhesion to the walls of the tooth. These I think are qualities indispensable to success. Still at this point we are met with favorable reports from the practice of others, who are in the habit of applying such caustic substances as the oxy-chloride of zinc and other strong corsives directly to the pulp, but I have not the temerity to use such agents in direct contact with the denuded tooth pulp.

I have already remarked that the correct theory of practice in these cases is simply protection. In adopting this theory we act upon the same course of practice with the general surgeon in the treatment of a denuded cerebrum or cerebellum, who seeks to protect simply the denuded organ—having first removed by excision, protruding and all greatly injured parts. To my mind there is a very striking parallelism between the two kinds of injury—they certainly differ more in magnitude than in kind.

In this connection, if you will allow the digression, I remark that I think it a mistake for the dentist to practice, in these cases with reference to the possible deposition of bone to close up the breach made into the pulp chamber by caries or other means. I think he should rather regard the substance placed for this purpose, by his own hand as a permanent necessity to the case—and thank nature for it, if she should be kind enough to come to his assistance in the case subsequently—which she certainly does occasionally—I am happy to know however that we are able to succeed without this extraordinary action on the part of nature.

In this article I have chosen to speak only of simple exposure, by which we understand exposure without either congestion or inflammation, and its treatment, because of the great length that must have been added to treat of all of the phases of exposure with congestion; with inflammation active and chronic; with inflammation, chronic and suppurative; exposure, with chronic suppurative inflammation, with protruding, strangulated pulp, etc. The general principles hinted at or outlined in simple cases is the same in all complicated ones, with excision cauterizations and medications, constitutional and local superadded.

